# Effects of tones associated with drilling activities on bowhead whale calling rates

**DOI:** 10.1371/journal.pone.0188459

**Published:** 2017-11-21

**Authors:** Susanna B. Blackwell, Christopher S. Nations, Aaron M. Thode, Mandy E. Kauffman, Alexander S. Conrad, Robert G. Norman, Katherine H. Kim

**Affiliations:** 1 Greeneridge Sciences, Incorporated, Santa Barbara, California, United States of America; 2 Western EcoSystems Technology, Incorporated, Cheyenne, Wyoming, United States of America; 3 Scripps Institution of Oceanography, University of California San Diego, La Jolla, California, United States of America; Pacific Northwest National Laboratory, UNITED STATES

## Abstract

During summer 2012 Shell performed exploratory drilling at Sivulliq, a lease holding located in the autumn migration corridor of bowhead whales (*Balaena mysticetus*), northwest of Camden Bay in the Beaufort Sea. The drilling operation involved a number of vessels performing various activities, such as towing the drill rig, anchor handling, and drilling. Acoustic data were collected with six arrays of directional recorders (DASARs) deployed on the seafloor over ~7 weeks in Aug–Oct. Whale calls produced within 2 km of each DASAR were identified and localized using triangulation. A “tone index” was defined to quantify the presence and amplitude of tonal sounds from industrial machinery. The presence of airgun pulses originating from distant seismic operations was also quantified. For each 10-min period at each of the 40 recorders, the number of whale calls localized was matched with the “dose” of industrial sound received, and the relationship between calling rates and industrial sound was modeled using negative binomial regression. The analysis showed that with increasing tone levels, bowhead whale calling rates initially increased, peaked, and then decreased. This dual behavioral response is similar to that described for bowhead whales and airgun pulses in earlier work. Increasing call repetition rates can be a viable strategy for combating decreased detectability of signals arising from moderate increases in background noise. Meanwhile, as noise increases, the benefits of calling may decrease because information transfer becomes increasingly error-prone, and at some point calling may no longer be worth the effort.

## Introduction

After summering in the eastern Beaufort Sea, the Bering-Chukchi-Beaufort (BCB) population of bowhead whales (*Balaena mysticetus*) begins its autumn westward migration in late August. Unlike the spring migration, the autumn migration takes place relatively close to the northern shores of Alaska [1]. Since the early 1970s, these areas have encountered various types of industrial activities associated with the oil and gas industry. For example, in the Canadian Beaufort alone about 90 wells have been drilled to date, using either drill ships or artificial islands [2].

Starting in 2006 and ending in 2014, an acoustic monitoring program was implemented by the Shell Exploration and Production Company (Shell) as part of their exploration activities in the Beaufort Sea. In summer 2012 Shell performed exploratory drilling at *Sivulliq*, a lease holding located about 30 km offshore near longitude 146° W, between Prudhoe Bay to the west and Camden Bay to the east (for more information, see [3]). Over the nine-year acoustic monitoring program, 2012 was the only year with drilling activities taking place within the study area, providing an opportunity to examine the effects of such activities on the behavior of migrating bowheads.

Early studies of the reactions of baleen whales to drilling operations, many of which were published in the gray literature, are summarized in detail in Richardson *et al*. [4]. Four of the studies mentioned, which took place in 1986 [5] and 1991–1993 [6–8] are particularly relevant to Shell’s drilling operations at *Sivulliq* in 2012, as these four studies pertain to bowhead whales migrating in the autumn past active drillships and support vessels in the Beaufort Sea. In 1986 and 1993, bowhead whales appeared to avoid an area of radius ~10 km around the drillship, deflecting either seaward or shoreward around the operation, with some whales apparently beginning to divert when 20 km or more from the drill site [5, 8]. Greene [9] reported that the average broadband sound levels at distance 10 km were ~114 dB re 1 μPa. In 1991 and 1992 ice was heavier, and most of the whales remained ~20 km or more seaward of the drilling operation. The authors were not able to determine whether the wider deflection was due to the drilling activities or constituted a normal response to heavier ice [6, 7].

To our knowledge, there is only one other study assessing effects of drilling activities on calling behavior in bowhead whales. Richardson *et al*. [10] performed playback experiments of drilling and dredging noises and observed bowhead reactions to these sounds. They reported behavioral reactions in most of the animals, such as orienting away from the sound, cessation of feeding, and altered SRD (surfacing, respiration, and diving) cycles. Calling rates may have decreased during playbacks, but no conclusive statement was made about this result because the authors were unable to determine how much of that decrease was due to masking by the projected sounds *versus* an actual decrease in calling rate.

Two previous studies [11, 12] have demonstrated that sounds from airgun pulses have measurable impacts on the calling rates of bowhead whales during their westward fall migration. Specifically, bowhead calling rates increased as soon as airgun pulses were detectable, but then decreased with higher received doses of airgun sound until there was no more calling. In the work presented here, we apply a similar methodology as that presented in Blackwell *et al*. [12], but use it to quantify changes in bowhead calling rates in response to industrial tonal noise generated by vessels and machinery during the 2012 drilling operation.

## Materials and methods

### Equipment

The equipment and field methods employed for this study are nearly identical to those presented in Blackwell *et al*. [12]. Recordings were made using DASARs (Directional Autonomous Seafloor Acoustic Recorders, [13]) sampling continuously at 1 kHz on three channels: one omnidirectional pressure channel and two particle motion channels. The omnidirectional channel included a calibrated hydrophone with a sensitivity of -149 dB re 1 V / μPa at 100 Hz, and a noise floor of 62 dB re 1 μPa^2^ / Hz @ 10 Hz, 48 dB @ 50 Hz, 44 dB @ 100 Hz, and 37 dB @ 400 Hz. This sensor was used for sound pressure measurements of any sounds of interest, including whale calls, distant airgun pulses, or various types of background noise. The directional channels included two particle motion sensors mounted orthogonally in the horizontal plane for sensing direction to sounds for which a bearing was required, such as whale calls and airgun pulses. DASARs include a signal digitizer with 16-bit quantization. The 1 kHz sampling rate allowed for 116 days of continuous recording in the frequency range 10–450 Hz.

### Field procedures

**Fig 1:**
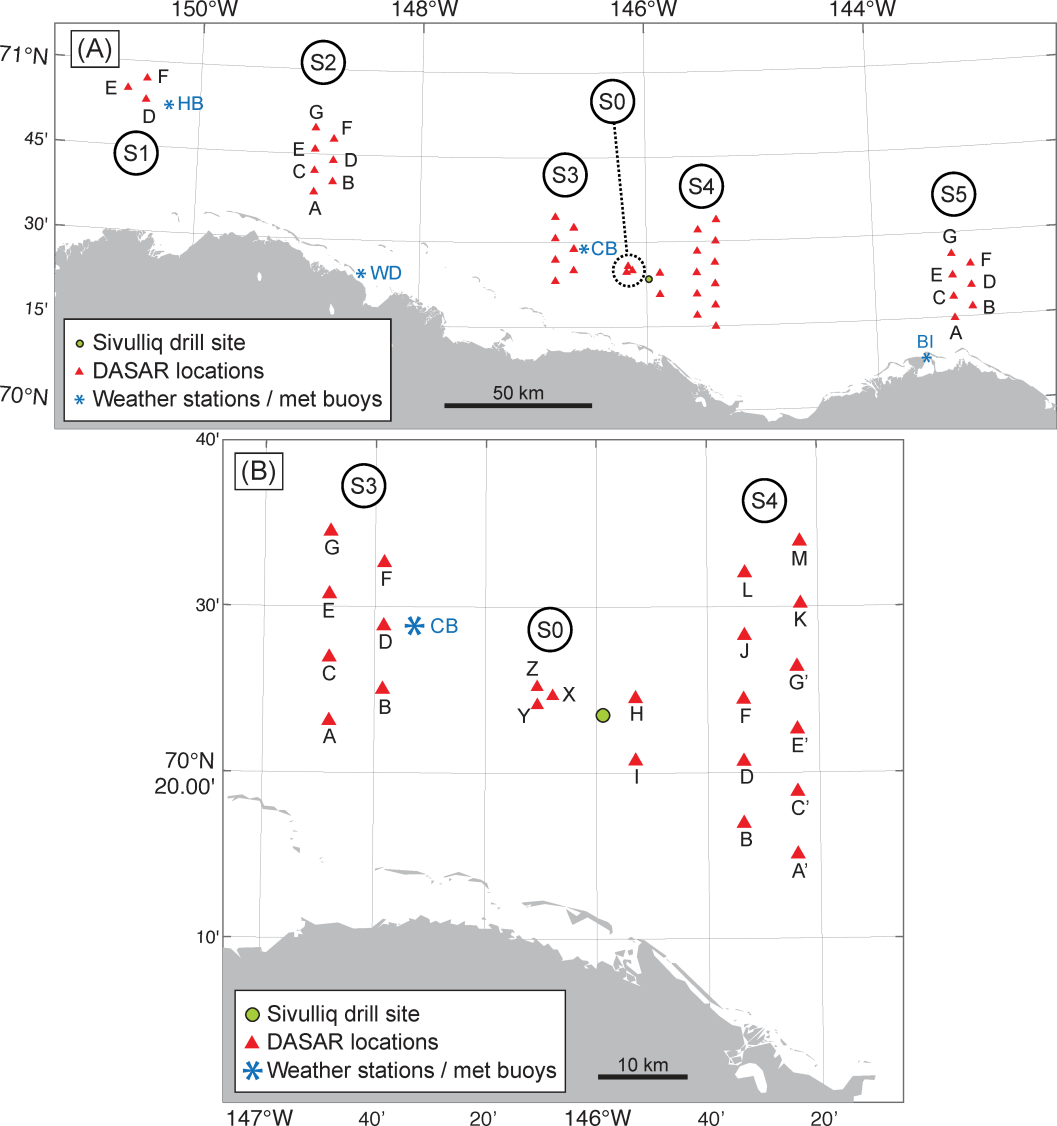
DASAR deployment locations. (A) Entire study area, showing the six arrays of recorders, with each array grouped into one of six “sites”, labeled S0 to S5. The east-west span (site 1 to site 5) is ~280 km. DASAR locations are shown as red triangles and a green dot shows Shell’s *Sivulliq* drill site. (B) Enlargement of the center of the study area, comprising the three sites closest to the drilling location: sites 3, 0, and 4. DASARs at each site are labeled with letters. Blue asterisks show the locations of two weather stations, West Dock (WD) and Barter Island (BI), and two meteorological buoys, Camden Bay (CB) and Harrison Bay (HB).

During the open-water season of 2012, 40 DASARs were deployed in the Beaufort Sea, in six arrays of 3–13 recorders each that spanned an east-west distance of ~280 km (Fig 1). In each array, DASARs were placed at the vertices of an equilateral triangular grid with 7 km sides. Exceptions included three DASARs at site 0, which were only separated by 2 km, and two DASARs at site 4, 4H and 4I, which were separated by 7 km but lacked the third member of the triangle (Fig 1). Compared to previous years (2007–2011, *e*.*g*., 11, 12), DASAR locations in 2012 were slightly changed, to better serve the purpose of industrial sound monitoring during Shell’s drilling operation. For example, the four northernmost DASARs at site 4 and the three site 0 DASARs were new locations for the 2012 field season. The approximate distance from the *Sivulliq* drilling location to the center of each array was 178 km for site 1, 114 km for site 2, 29 km for site 3, 7 km for site 0, 19 km for site 4 (but with a wide spread, from 4 km for 4H to 30 km for 4M), and 103 km for site 5.

DASARs were deployed between 16 and 21 August and retrieved between 3 and 7 October 2012. Each DASAR was placed on the seafloor with a 110-m ground line connecting it to a small Danforth anchor, and GPS positions of the recorder and the anchor were obtained on each deployment. DASARs were then retrieved by grappling for the ground line. The S1 Table (supporting information) shows deployment and retrieval dates, deployment locations, and water depths for all forty instruments.

Immediately after deployment and preceding retrievals, each DASAR’s clock and orientation on the seafloor were calibrated by transmitting specific signals (frequency range 200–400 Hz, source level ~150 dB re 1 μPa) at known GPS-determined times and locations, three around each recorder. For more information on the calibration methodology, see [13, 14].

### Permitting

Passive acoustic recording of endangered bowhead whale calls does not typically require a federal permit as it does not have the potential to “take” the animals as defined by the U.S. Marine Mammal Protection Act or the U.S. Endangered Species Act. The research presented here was, however, part of an approved monitoring program around activities conducted under incidental harassment authorizations (IHAs) issued by the U.S. National Marine Fisheries Service. The research was therefore subject to regulatory review and approval under those authorizations and under the terms of lease agreements under the Outer Continental Shelf Lands Act.

### Analysis strategy

The analysis strategy used here is similar to that used in Blackwell *et al*. [12]. The entire field season was split up into short time intervals (10-min, see below), and for each of these time intervals at each DASAR, data for three variables were collected: the levels of two specific types of industrial sound, *i*.*e*., airgun pulses and tones from machinery (the “dose” in a dose-response study), and the number of whale calls detected (the “response” in a dose-response study). Sound from airgun pulses was quantified because many pulses were present in 2012, and previous work had demonstrated that these signals have measurable effects on bowhead calling rates [12]. Tones were quantified because they are a good indication of the presence of machinery and vessels and, unlike many other components of industrial sound, they can be identified and separated from wind-driven background noise. Using negative binomial regression, various models were then fit to the data, and the best model was selected based on its Bayesian Information Criterion (BIC) [15].

### Time intervals

The analysis required defining the length of the time interval over which the dose (industrial sound) and response (whale calling rate) would be matched. The interval needed to be long enough that many intervals included at least one call, and it needed to be relevant for whale response to received levels of sound. Based on these considerations as well as previous analyses [12], we chose a time interval of 10 minutes. The entire 2012 field season was divided into non-overlapping, 10-min periods that always began on minute 0, 10, 20, 30, 40, or 50 of each hour.

### Detection and localization of bowhead calls

After retrieval, data were transferred to file servers and analyzed using custom MATLAB-based software. An automated call detection algorithm [16] was applied to the data collected at each site. During whale call analysis, bearings to calling whales were determined for every detected call. When a call was detected by at least two DASARs, the location of the whale was estimated using triangulation [13, 16]. Since we only considered localized calls in this study, we define “call localization rate” simply as a calling rate limited to localizable calls. Calls detected on just one DASAR are thus not included in the analysis.

### Defining the analysis area

This study aims to identify if a relationship exists between received levels of sound from tones and the calling behavior of bowhead whales. It is therefore of critical importance that all calls included in the analysis have approximately the same probability of being detected. Blackwell *et al*. [12] faced a similar problem and determined that by restricting samples to whale calls that were within a two-km radius of a DASAR, mean detected call rates did not decrease with increasing levels of background sound, *i*.*e*., were unaffected by masking (see Fig A of the S2 File in [12]). In that study, however, the sounds of interest were airgun pulses, which are fired intermittently. In the present study, sound sources were generally continuous, and remained on for a minimum of minutes, and often many hours. At recorders that were closest to the drilling operation (*e*.*g*., site 0 and locations 4H and 4I, see Fig 1B) and during certain specific high-amplitude activities (such as anchor setting, see [3]), received levels were high enough for the DASAR hydrophone to reach saturation, sometimes over several consecutive hours. Therefore, no circle radius would be small enough to eliminate the masking problem.

**Fig 2:**
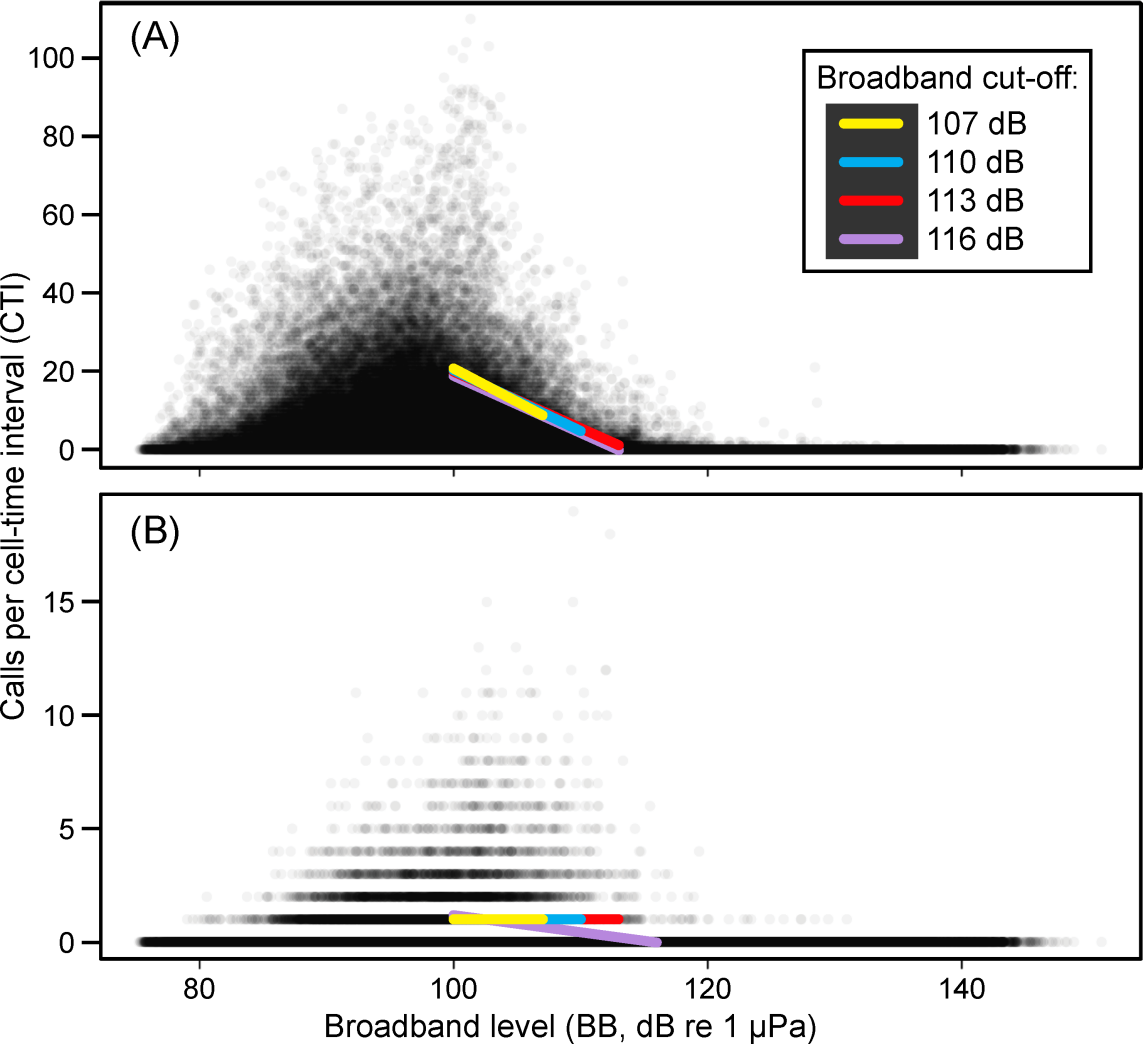
Relationship between background levels *BB* and call localization rates (calls / 10 min). (A) All localized calls; (B) Only calls within 2 km of a DASAR. Dots are displayed as 95% transparent. The colored lines show the predicted 97.5^th^ percentile of number of calls based on quantile regression, for the four possible cut-off values: 107 dB (yellow), 110 dB (turquoise), 113 dB (red), and 116 dB re 1 μPa (lavender). The lower cut-off was set at 100 dB in all cases, to focus on sound levels where masking might occur, see text. Line equations are shown in the S2 Table. Note that the vertical axis is much expanded in (B) compared to (A).

To address this issue, we took a two-step approach: (1) 10-min periods with a mean broadband (background) level (*BB*, see below) above 113 dB re 1 μPa were removed from the analyses (see below), and (2) for the remaining 10-min periods, *i*.*e*., with mean background levels below the 113 dB cut-off, only calls localized within two km of a DASAR were included in the analyses. Hereafter, a particular analysis cell (2-km circle) at a particular 10-min time interval will be referred to as a “cell-time interval” (CTI). Fig 2 illustrates how the two-step approach explained above resulted in a dataset in which detection probabilities for calls were near-constant.

Broadband levels were calculated for every minute at every DASAR over the entire deployment period. Narrowband spectral densities (1 Hz bin width, 1.7 Hz noise bandwidth) were determined for each minute of collected data by analyzing a series of 119 one-second-long data segments, overlapped by 50%, and then averaging them. Broadband levels were derived from the narrowband spectral data by integrating the mean square pressures of all frequencies within the 10–450 Hz frequency range. A mean broadband level *BB* was determined for each 10-min time interval at each DASAR by averaging the one-min broadband values in the linear domain.

Fig 2 shows the number of bowhead calls detected as a function of the mean broadband level *BB*, when (A) using all calls localized versus (B) using only calls localized within 2 km of a DASAR. The four lines show the 97.5^th^ percentile of number of calls based on quantile regressions, for *BB* greater than 100 dB (the lower cut-off) and four alternative upper cut-offs: 107 dB, 110 dB, 113 dB, and 116 dB re 1 μPa. Several alternative cut-offs were tested since our goal was to use as much of our dataset as possible while still satisfying the constraint of detected calling rates being independent of background levels. Since interest focused on masking, which we had no reason to suspect at the lowest broadband levels, two lower cut-offs were tested, at 95 dB and 100 dB (the 100 dB choice is shown in Fig 2). The choice of the 97.5^th^ percentile was driven by the fact that the data were dominated by zeros, particularly for the (B) subset. Between each pair of cut-offs (each combination of lower and upper cut-offs, for the (B) subset), there were no calls in 95.4–96.7% of all CTIs. Therefore, using a lower percentile, say, the 95^th^ percentile (or any lower value) would have been uninformative.

The S2 Table shows the regression equations and confidence intervals for each of the 16 lines generated from using two lower cut-offs, four upper cut-offs, and two call subsets ((A) and (B) above). Section B.2 in the S2 Table shows that when restricting the dataset to calls localized within 2 km of a DASAR and using a lower cut-off of 95 dB, all lines had confidence intervals on the slope that included a slope of 0, *i*.*e*., indicating no relationship between background levels and call detection rates. Section B.1 (S2 Table) shows that when the 100-dB lower cut-off was used, only the 116-dB upper cut-off had a non-zero slope. We therefore settled on using an upper *BB* cut-off of 113 dB re 1 μPa.

In summary, above broadband levels of 100 dB in Fig 2A, there is a clear negative relationship between broadband levels and call localization rates, indicating that masking would affect our analysis since localization rates of all calls decrease as broadband levels increase. This relationship has been eliminated in Fig 2B, when only calls localized within 2 km of a DASAR are considered.

The entire dataset contained 273,522 cell-time intervals (CTIs). At site 0, the DASARs were only 2 km apart instead of 7 km, so to avoid duplicate whale counts at overlapping CTIs, only the data for DASAR 0Y (chosen randomly) were included in the analyses. This decreased the number of CTIs to 258,617. The 113 dB *BB* cut-off then removed a further 35,130 CTIs (14% of the dataset), listed as follows by site (from west to east):

- 0.5% for site 1 (88 out of 18,246 cell-time intervals)
- 1.9% for site 2 (898 out of 46,996)
- 23% for site 3 (10,519 out of 45,795)
- 27% for site 0 (1,981 out of 7,458)
- 22% for site 4 (20,777 out of 93,906), and
- 1.9% for site 5 (867 out of 46,216).

The higher percentages at sites 3, 0, and 4 were expected, since those sites are closest to the *Sivulliq* drilling location (Fig 1). Once usable CTIs had been selected, the numbers of localized calls within 2 km of each DASAR were tallied for the analysis dataset. Note that calls that were localized within 2 km of a DASAR but not detected by that DASAR were excluded.

### Industrial sound indices

A large-scale drilling operation in the Arctic involves a number of ships: the drill ship itself, towing vessels, ice management vessels, resupply vessels, crew change vessels, and oil spill response vessels, all using various types of sound-producing machinery (Shell’s 2012 drilling effort involved a particularly large number of vessels due to regulatory mandate). A previous study [12] investigated the effects of airgun pulses on the calling behavior of bowhead whales. Airgun pulses are relatively easy to detect and the sound they produce is relatively easy to quantify. A drilling operation, on the other hand, consists of numerous continuous sound sources that cannot readily be separated from natural background noise. We therefore sought to quantify these industrial sounds with the use of a metric, defined here as “*Tones”*, which is correlated with the overall levels of continuous industrial noise.

#### Tones index

**Fig 3:**
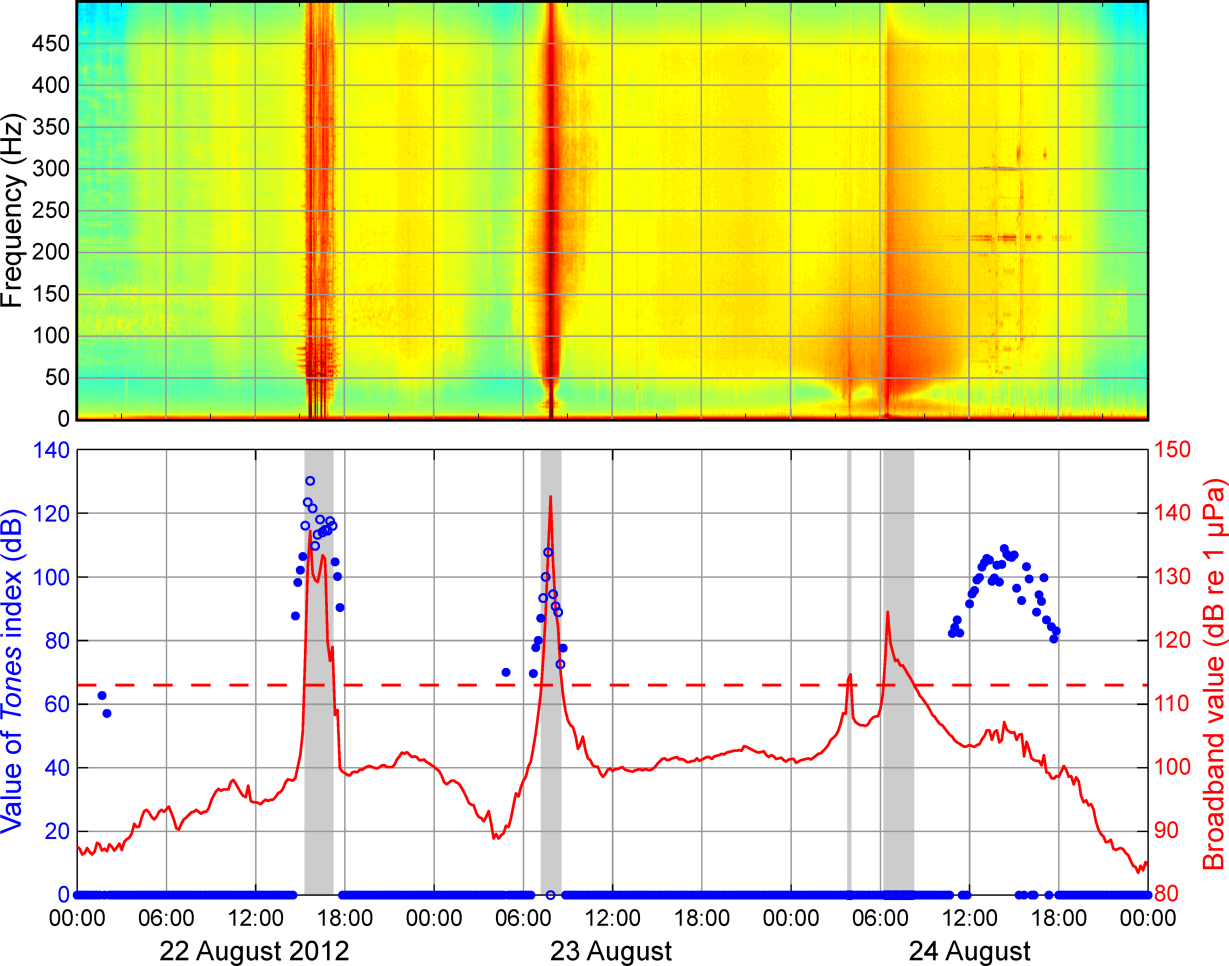
Broadband levels and values of the *Tones* index at DASAR 2D, 22–24 August 2012. The top panel shows a spectrogram of the DASAR data over the same period. Tones are visible in the spectrogram as red horizontal lines. In the lower panel, blue dots and circles are *Tones* values for cell-time intervals (10-min periods) at the DASAR. When no tones were detected, *Tones* = 0. The solid red line is the broadband value *BB*. Cell-time intervals with *BB* > 113 dB (dashed red line) were excluded from the analysis. *Tones* detections that occurred during such periods—as indicated by gray shading—were therefore excluded from the analyses, and are shown with blue circles. *Tones* detections during periods with *BB* < 113 dB (blue dots) were retained for analysis.

This index quantifies the intensity of tones that are present in the sound spectrum, relative to the complete background noise intensity. We define a tone as a sound of bandwidth 1 Hz with an amplitude at least 4 dB above that of neighboring frequencies and that occurs continuously over several minutes (see the S1 File). Tones are typically produced by machinery, such as vessel engines, generators, drilling equipment, etc., and are therefore characteristic of industrial sound. For each two-min period of DASAR data, the metric identified the tones present, integrated their total intensity and related this intensity to a total background level, averaged over the two minutes. This quantity, similar in concept to a signal-to-noise ratio (and therefore unitless), provides a measure of the relative contribution of tonal sound to the total background noise field for each two-min period. The *Tones* index for a 10-min cell-time interval was obtained by summing the two-min values over 10 min. Details of how the *Tones* index was calculated, with an example, are presented in the S1 File. Fig 3 illustrates values taken by the *Tones* index over a period of three days with variable sound levels at DASAR 2D.

#### Airguns index

Airgun pulses were present on all 40 DASAR records during the 2012 field season. Since airgun pulses were known to affect bowhead whale calling rates [12], they also needed to be taken into account. In Blackwell *et al*. [12], the sound received from airgun pulses was quantified as a cumulative sound exposure level (CSEL) calculated over 10 min. CSEL values varied from less than 80 dB to more than 175 dB re 1 μPa^2^-s. In 2012, two different seismic exploration operations were identified, to the east and to the north of the DASAR arrays [17]. Both operations were relatively distant, up to 100s of km from the DASARs, resulting in received levels (RLs) of airgun sound that were much lower than those in Blackwell *et al*. [12]. In 2012, 95% of 10-min CSELs were below 130 dB and no values exceeded 140 dB re 1 μPa^2^-s. Since this restricted range of RLs from airgun pulses would not have allowed us to replicate the results of Blackwell *et al*. [12], we used a simple indicator variable, called ***Airguns***, to describe the presence (1) or absence (0) of detected airgun pulses in each 10-min CTI. Airgun pulses were detected, analyzed, and the detections were quality-controlled as described in Blackwell *et al*. [12].

### Modeling of whale call numbers as a function of received levels of industrial sound

Covariates considered in the negative binomial regression modeling included the two sound metrics (*Tones* and *Airguns*) and *Site* (represented by five indicator variables for the six sites). We checked for potential collinearity among the candidate covariates using condition indices and variance decomposition proportions [18]. These tests indicated that multi-collinearity among the covariates was not an important issue.

In constructing models, we considered categorical *Airguns*, linear, quadratic, and cubic effects for *Tones*, categorical *Site*, and any potential interactions between each of the sound measures and *Site*. Models were constructed with the restrictions that models with higher order effects (quadratic or cubic) necessarily included the corresponding lower order effects, and models with interactions included the corresponding main effects. Thus, models included the main effects of *Site*, *Tones*, and *Airguns*, with corresponding higher order effects and interactions, for a total of 29 candidate models. We evaluated models based on relative fit (BIC, [15]).

To address potential spatio-temporal correlation in whale call counts and its effects on parameter variances, we conducted block bootstrapping [19]. These procedures (see the S2 File) allowed us to calculate 95% confidence intervals for the regression model parameters without making explicit distributional assumptions.

## Results

### Dataset

The analysis dataset, *i*.*e*., after removal of CTIs with *BB* > 113 dB re 1 μPa, included 223,487 cell-time intervals and 14,751 bowhead calls (Table 1, Section (A)). The two easternmost sites, sites 4 and 5, included 53% of all CTIs and 56% of all whale calls.

**Table 1:** Summary of cell-time intervals (CTIs) and whale calls at different sites. Sites are listed from west (site 1) to east (site 5). Section (A) summarizes the number of CTIs and total number of whale calls detected at each site in the analysis dataset (i.e., after removal of CTIs with BB > 113 dB re 1 μPa). Sections (B), (C), and (D) compare the expected versus actual percentages of calls in CTIs with detected tones (B), airguns (C), and both tones and airguns (D). For example, at site 1, 7.9% of the 18,158 site 1 CTIs included tones, and 13.6% of the 894 site 1 whale calls were detected in those CTIs. Therefore, when tones were present the “excess” calls at site 1 was 13.6%– 7.9% = 5.7%. In (D), CTIs with both tones and airguns are compared to CTIs with neither tones nor airguns.

		Site 1	Site 2	Site 3	Site 0	Site 4	Site 5	SUM
**A.**	**CTIs:**	18,158	46,098	35,276	5,477	73,129	45,349	223,487
	As a %:	8.1	20.6	15.8	2.5	32.7	20.3	100
	Calls:	894	2340	2781	421	3347	4968	14,751
	As a %:	6.1	15.9	18.9	2.9	22.7	33.7	100
**B.**	**TONES**							
	% of CTIs	7.9	23.7	24.8	32.6	29.8	15.3	
	% of calls	13.6	36.2	38.0	23.3	36.9	33.9	
	Excess	**5.7**	**12.5**	**13.2**	**-9.3**	**7.1**	**18.6**	
**C.**	**AIRGUNS**							
	% of CTIs	5.4	34.6	38.8	36.5	50.9	78.5	
	% of calls	7.2	51.2	52.1	69.8	72.3	83.3	
	Excess	**1.8**	**16.6**	**13.3**	**33.3**	**21.4**	**4.8**	
**D.**	**BOTH**							
	% of CTIs	1.6	17.5	18.9	17.3	28.7	40.1	
	% of calls	2.2	38.7	41.4	41.3	60.5	71.5	
	Excess	**0.6**	**21.2**	**22.5**	**24.0**	**31.9**	**31.4**	

Whale calls were not spread evenly between sites, or between CTIs receiving different types of industrial sound. Sections (B)–(D) of Table 1 show the percentage of cell-time intervals that included tones (Section (B)), airguns (Section (C)), or both tones and airguns (Section (D)), for each of the six sites. For example, Section (B) shows that CTIs with detected tones made up 15.3% of the CTIs at site 5. If whale calls occurred at the same rate regardless of site or sound sources, then 15.3% of whale calls (at site 5) should have occurred during the CTIs with tones, and not 33.9% as shown in Section (B). We call this difference between the actual and expected percentages (33.9–15.3 = 18.6%) the “excess” calls. Section (B) shows that all sites but site 0 had an excess of calls when tones were present.

Similarly, Section (C) shows that when airgun pulses were present, all sites showed excess calls (range 2–33%), even though the excess was modest for sites 1 and 5. Section (D) compares CTIs with both tones and airguns with CTIs lacking detections of either sound source. Excess calls were present in the range 21–32% at all sites but site 1, where there was no difference.

**Fig 4:**
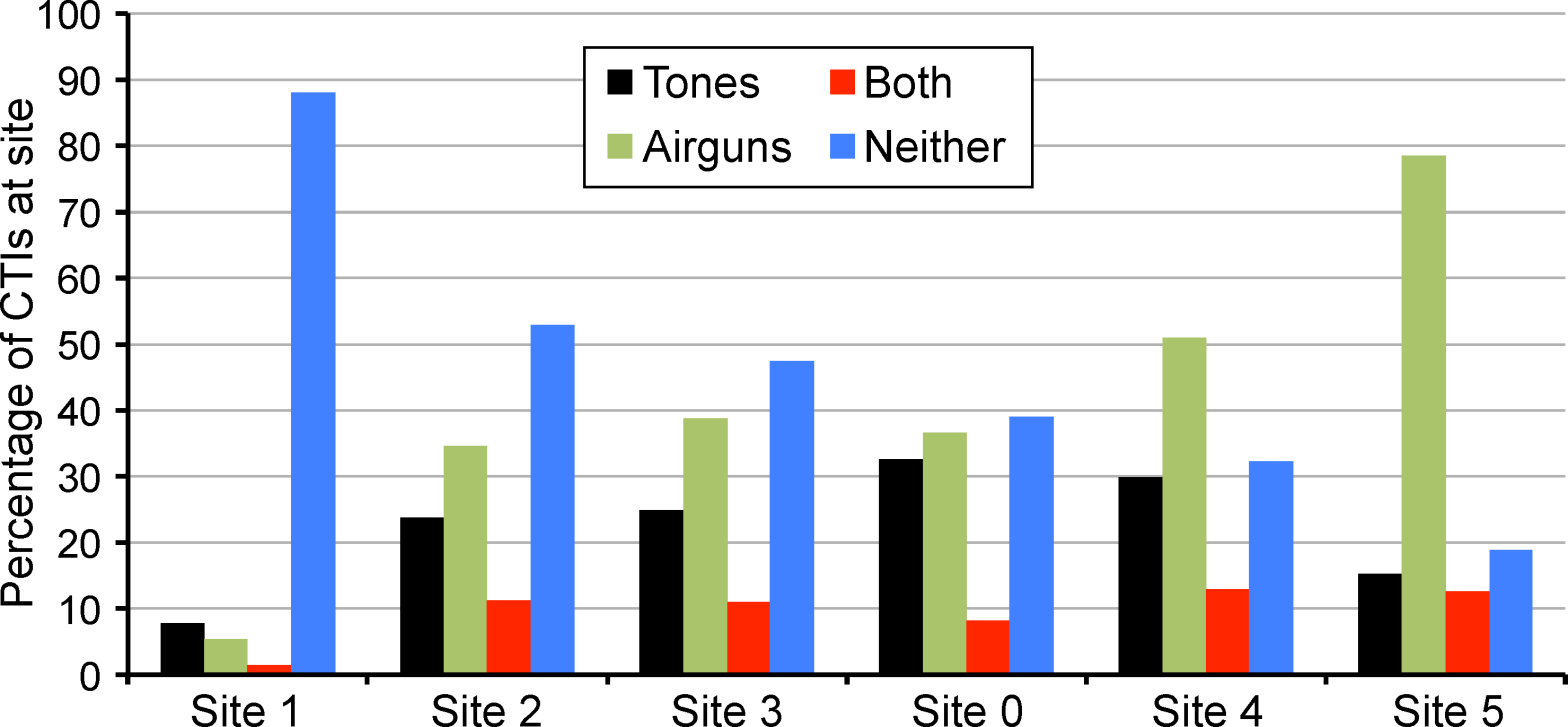
Percentages of cell-time intervals (CTIs) with detected tones and / or airguns. Only CTIs in the analysis dataset (Table 1(A)) are included. CTIs with tones detected (but no airguns) are shown with black bars, green bars show presence of airguns only, red bars show presence of both tones and airguns, and blue bars show neither.

Fig 4 summarizes the percentages of CTIs at each site for which (1) tones were present (*Tones* > 0), (2) airguns were present (*Airguns* = 1), (3) both tones and airguns were present, and (4) neither tones nor airguns were detected in the sound records (*Tones* = 0 and *Airguns* = 0). It shows that the percentage of CTIs with detected airguns increased from west (site 1) to east (site 5, see Fig 1). This is expected since the seismic operation to the east of site 5 ran almost continuously during the entire season [17]. In addition, mean site depth increased from west to east (Fig 1), and DASARs located in deeper water always detected more airgun pulses than their shallower counterparts. Fig 4 also shows that site 0, the site closest to *Sivulliq*, had the highest percentage of CTIs with detected tones. This percentage decreased for other sites east and west, as a function of distance from *Sivulliq*. As a result of these trends pertaining to the presence of tones and airguns, Fig 4 also shows that the percentage of CTIs with neither tones nor airguns detected decreased steadily from west to east.

### Negative binomial regression modeling

The 28 regression models assessing the relationship between the sound metrics and call localization rate had 2–30 parameters and BIC values ranging from 93,515 to 95,979. The S3 Table (supplementary material) summarizes all the models for the dataset. The two highest ranked models included *Site*, *Airguns*, and the cubic effect of *Tones* (*i*.*e*., the linear, quadratic, and cubic terms for *Tones*). In addition, the highest ranked model included the interaction between *Site* and *Airguns*, which resulted in it having 50% more parameters (15 *vs*. 10) than the second ranked model, with a modest advantage in BIC (ΔBIC = 16.1). Parameter estimates and associated 95% confidence intervals are listed in Table 2 for the highest-ranked model.

**Table 2:** Estimated coefficients and confidence limits for parameters in the chosen negative binomial regression model (model 1 in the S3 Table). L95 and U95 represent the lower and upper 95% confidence limits, respectively, estimated via block bootstrapping. Site 5 was the reference site; thus, it does not appear as a parameter in the model.

Parameter	Point estimate	L95	U95
Intercept	-2.626427	-3.215705	-1.757977
*Site 0*	-0.534597	-1.790348	0.256966
*Site 1*	-0.470289	-1.526295	0.379213
*Site 2*	-0.792156	-1.798242	0.090757
*Site 3*	-0.312135	-1.600178	0.703759
*Site 4*	-1.109251	-1.808653	0.071072
*Airguns*	0.277104	-0.050889	0.704608
*Tones*	-0.125023	-0.184340	-0.064801
*Tones*^*2*^	0.008796	0.005958	0.011547
*Tones*^*3*^	-0.000125	-0.000156	-0.000091
*Site 0 x Airguns*	0.911318	0.329954	1.402623
*Site 1 x Airguns*	-0.028275	-0.753625	0.868430
*Site 2 x Airguns*	0.322248	-0.220492	0.910640
*Site 3 x Airguns*	0.147567	-0.375442	0.908221
*Site 4 x Airguns*	0.555042	-0.173423	0.994211

Figures illustrating the modeling results are presented below. Figures that summarize the actual data are shown in a gray color scheme, with bar height indicating mean calling rate and error bars (± one standard error) showing variability in the data. In contrast, multi-colored plots are marginal effects plots based on the best regression model. These plots show the predicted calling rates as functions of model parameters (*Site*, *Airguns*, and *Tones*). It is important to keep in mind that when presenting modeled data in marginal effects plots, we assess the effect of any single covariate by holding other covariates at fixed values, as this helps in interpretation of the coefficient estimates.

**Fig 5:**
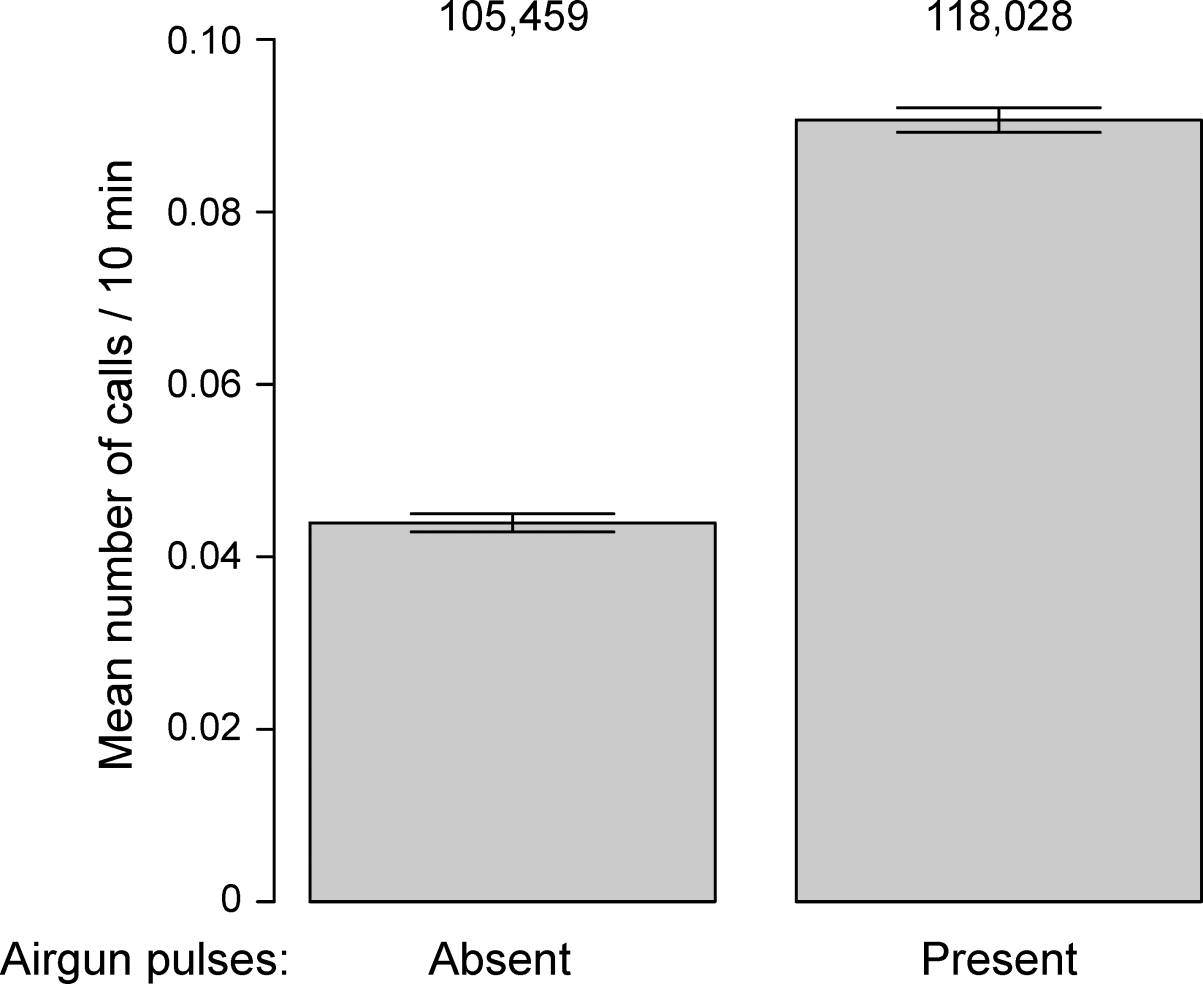
Mean calling rates with and without detected airgun pulses. Data are shown for all sites combined, ± one standard error, as a function of whether airguns were present (*Airguns* = 1) or absent (*Airguns* = 0), and irrespective of the value of *Tones*. Sample sizes are shown above the bars.

We emphasize that the gray-shaded plots are used for descriptive statistics only; no formal statistical inference (e.g. ANOVA) is directly associated with these bar charts, including whether mean calling rate is significantly greater when airgun pulses are present than when absent (see Fig 5). Rather, the statistical hypothesis testing is applied to regression coefficients obtained from fitting predictor variables (two noise metrics and geographic location) to the dependent variable (call localizations within 2 km radius per 10-minute interval). Our inferences are based on the terms in the selected model, which can be compared and contrasted with terms in the alternative models (S3 Table). As one example, the comparisons show how *Tones* consistently displays a non-linear relationship (cubic term) to call localization rate in the top four models, and that the inclusion of this cubic term generates better fits (lower BIC values) than comparable models in which *Tones* appears as only a linear or quadratic effect (*e*.*g*., in the S3 Table, compare Model 1 with Models 5 and 17, or compare Model 2 with Models 6 and 18).

#### Airguns

**Fig 6:**
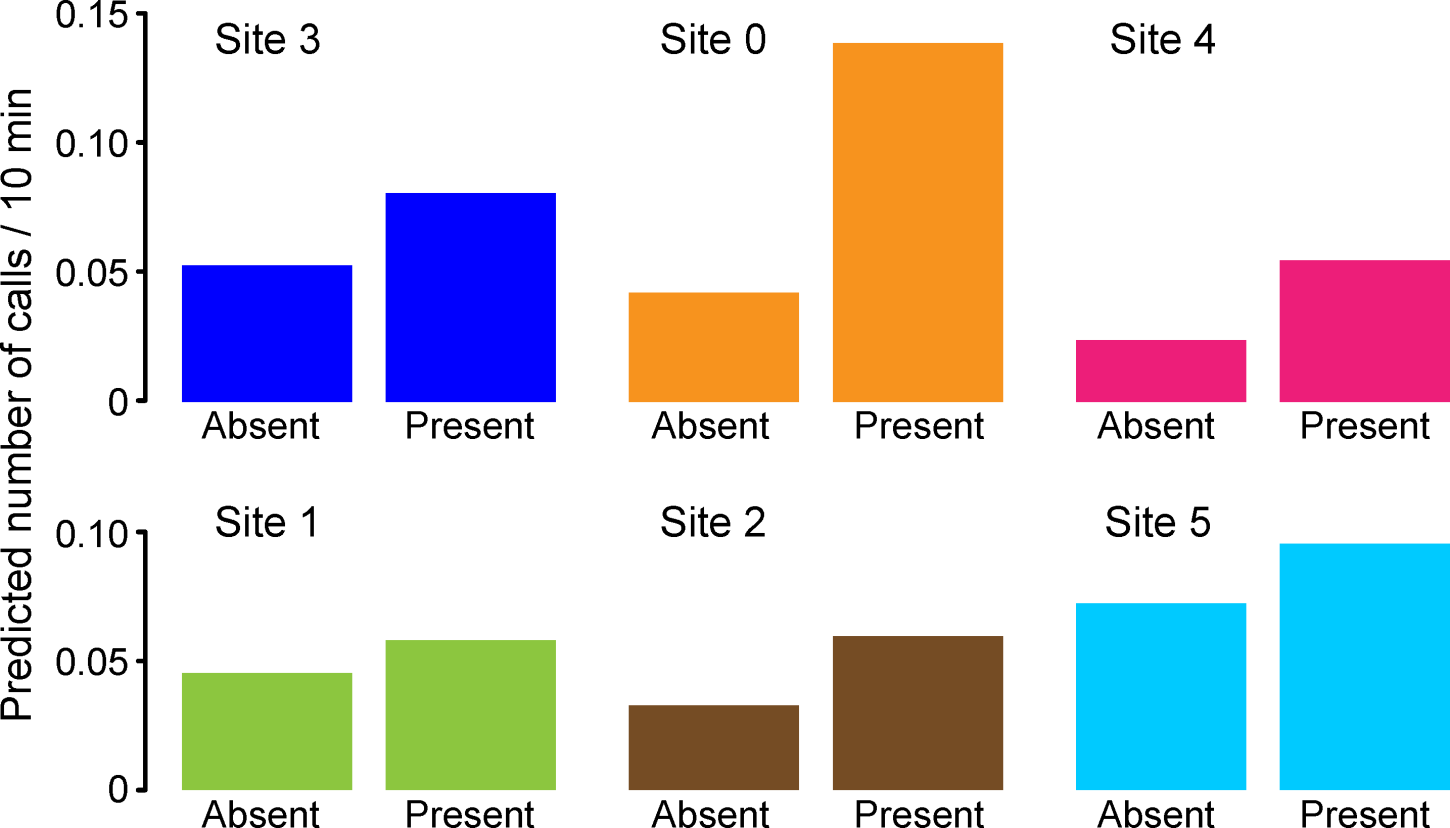
Calling rates predicted by the top model, with and without detected airgun pulses. Data are shown separately for each site, “absent” and “present” refer to airgun pulse detections, and the value of *Tones* is held at 0. Sites are shown from west (left) to east (right). The three sites in the top row are those relatively near the *Sivulliq* drilling location, with approximate distances of 29 km, 7 km, and 19 km to each array’s center, respectively. The bottom three sites were more distant, with approximate distances of 178 km, 114 km, and 103 km to each array’s center, respectively.

Observed calling rates in the presence and absence of airgun pulses, for the combined sites, is shown in Fig 5. Calling rates were on average 0.044 and 0.090 calls / CTI when airgun pulses were absent *vs*. present, respectively (all sites combined). The predicted number of calls at each site as a function of airgun presence is depicted in Fig 6 for the top model, with the *Tones* metric set to zero (*Tones* absent). These figures show that predicted calling rates increased in the presence of airgun pulses, but the size of the increase was site-dependent, with evidence of proportionately larger increases at the sites closer to *Sivulliq*.

#### Tones

**Fig 7:**
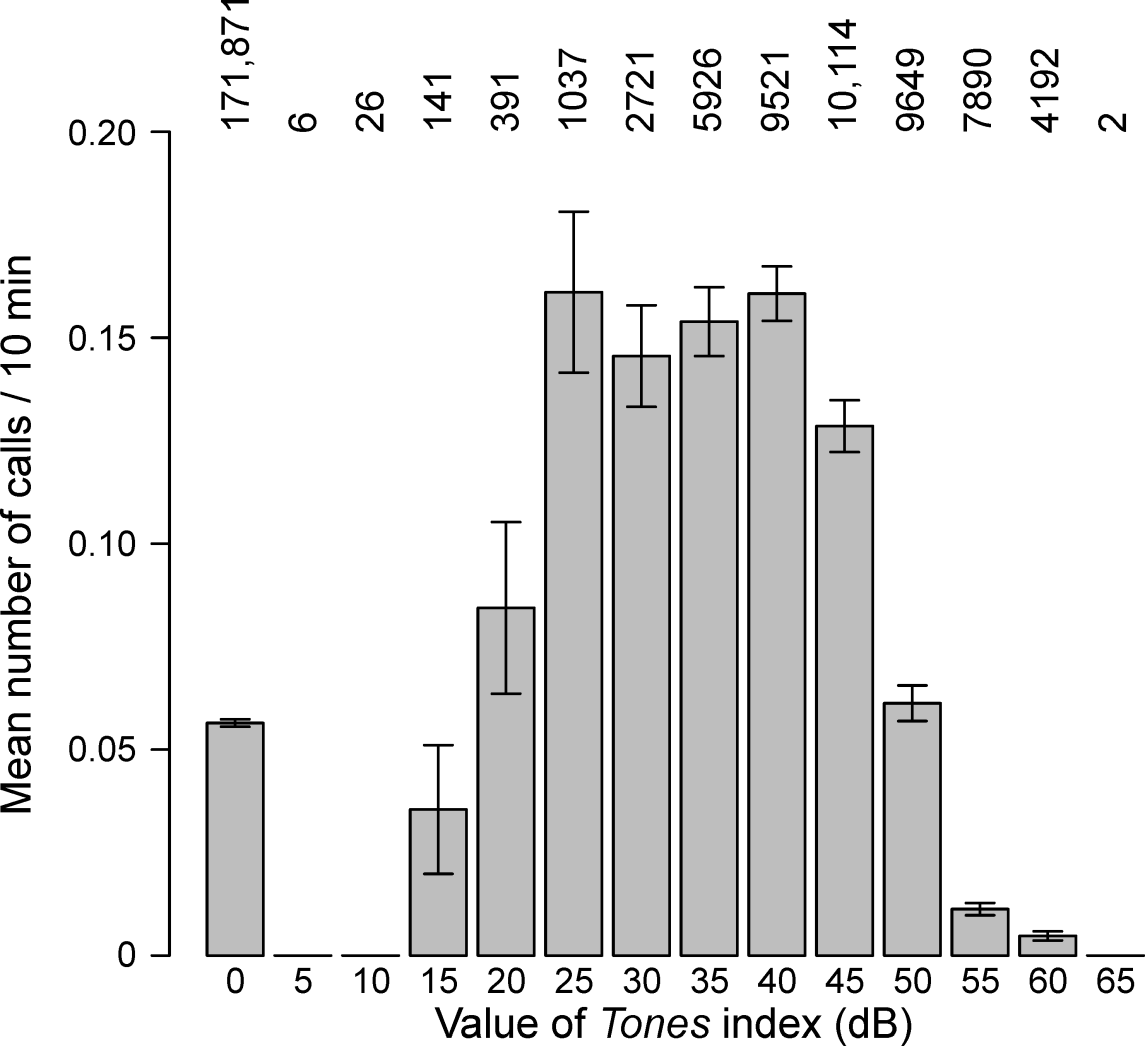
Mean calling rates at all sites combined as a function of the value of *Tones*. When *Tones* = 0, no tones were detected. Bars are shown ± one standard error with sample sizes above the bars. The value of *Airguns* was not taken into account. *Tones* is a unitless metric (see the S1 File for details).

Observed calling rates as a function of the presence and relative “dose” of tones is shown in Fig 7 for the combined sites. Compared to times when no tones were detected, increasing values of the *Tones* index initially lead to increased calling by the whales, but as the value of *Tones* continued increasing, calling rates decreased. In the absence of tones, calling rates were on average 0.056 calls / CTI (Fig 7). In comparison, when tones were present, average calling rates (across all *Tones* values and all sites) had increased to 0.098 calls / CTI. Furthermore, at the peak shown in Fig 7, corresponding to *Tones* bins in the range 25–40, the average calling rate was 0.156 calls / CTI.

**Fig 8:**
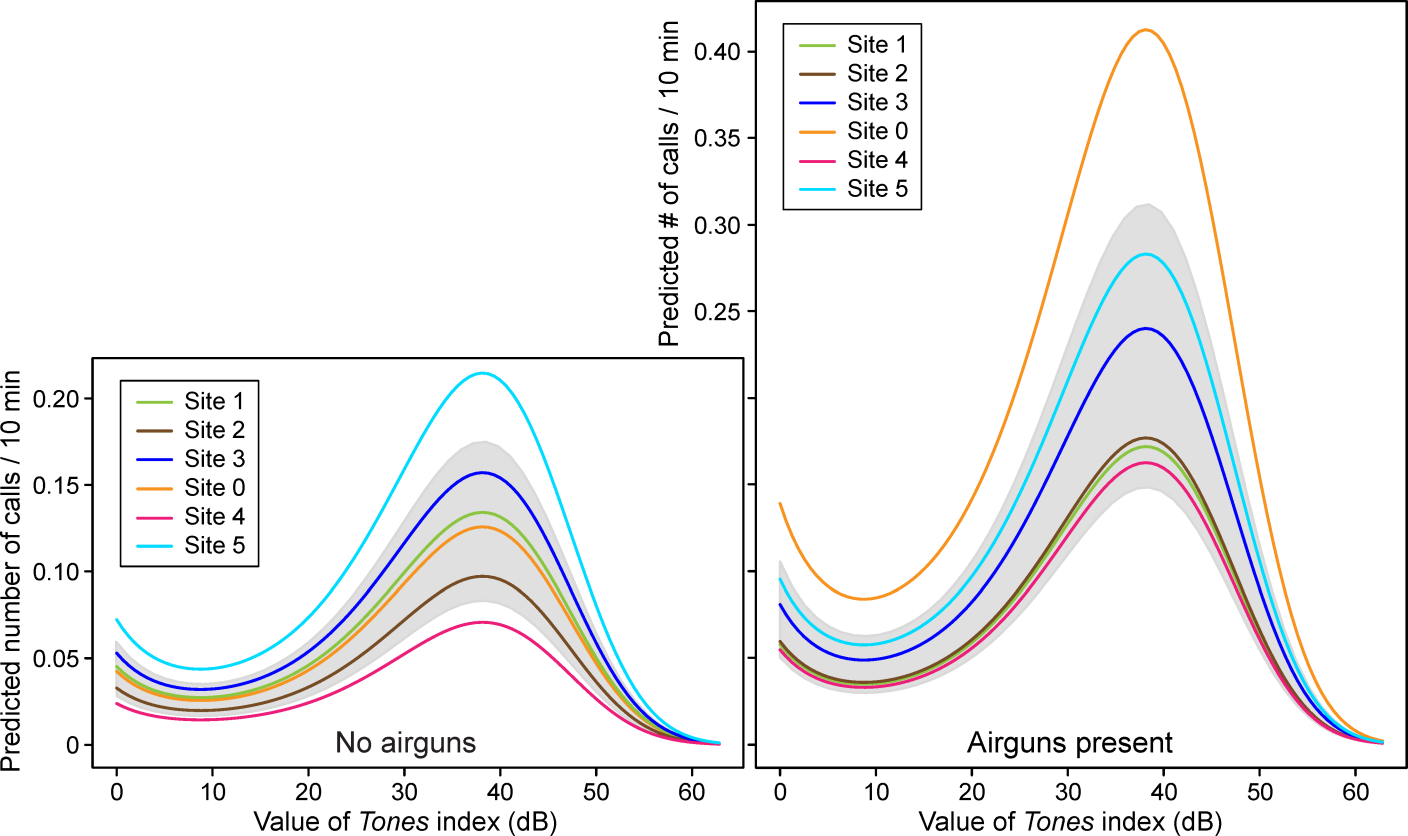
Calling rates predicted by the top model as a function of the value of *Tones* and the presence of airgun pulses. The left plot shows predicted calling rates in the absence of detected airgun pulses and the right plot shows the same information in the presence of airgun pulses. Each site is depicted in a different color. As a comparison, the gray shaded areas depict the range of values taken by the second ranked model (see the S3 Table, supplementary material), which was similar to the top model. *Tones* is a unitless metric (see the S1 File for details).

These trends are confirmed in the modeling results, shown for the top model in Fig 8 for each site, for CTIs with and without airguns. In the absence of airguns (Fig 8, left plot), predicted call localization rates were higher when tones were present, and increased with the value of *Tones* up to a *Tones* value of ~38 dB, beyond which they dropped precipitously. Peak predicted calling rates were highest at site 5 and lowest at site 4, with intermediate values for the other sites. When airguns were present (Fig 8, right plot), predicted calling rates were higher at all sites and at all but the highest values of *Tones* (>55 dB), where calling rates were near 0.

When airguns were present (Fig 8, right plot), site 0 surpassed site 5 as the site with peak calling rates, while the lowest calling rates were still predicted for site 4. Fig 8 also shows the range of values taken by the second ranked model (gray shading), confirming the similarities in the top two models.

#### Combined effects of tones and airguns

**Fig 9:**
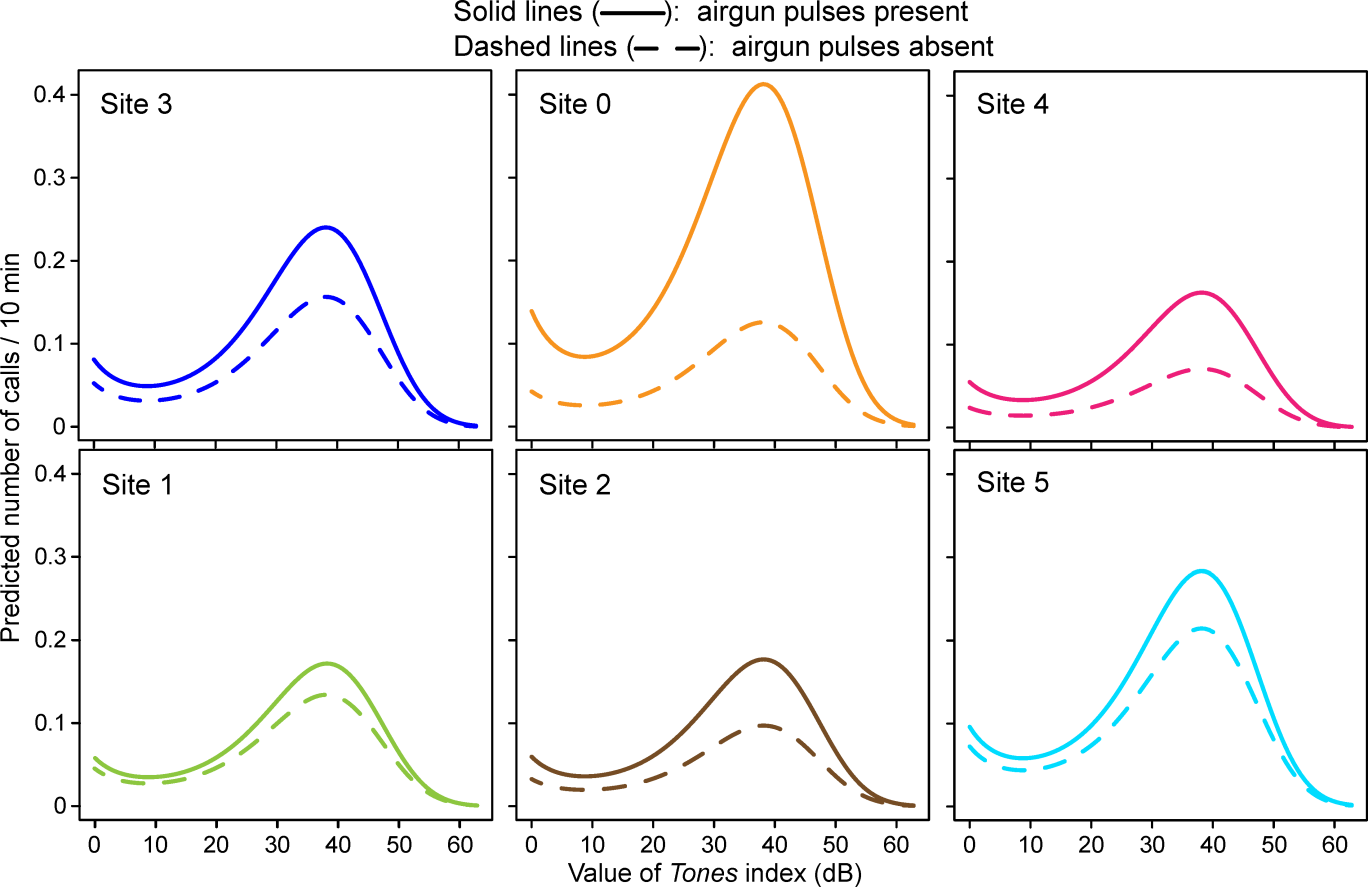
Predicted calling rates as a function of the value of *Tones*, with and without airgun pulses. Presence or absence of airgun pulse detections is shown with the solid or dashed lines, respectively. The six sites are shown separately, from west (left) to east (right). The three sites in the top row are those relatively near the *Sivulliq* drilling location, with approximate distances of 29 km, 7 km, and 19 km to each array’s center, respectively. The bottom three sites were more distant, with approximate distances of 178 km, 114 km, and 103 km to each array’s center, respectively. *Tones* is a unitless metric (see the S1 File for details).

Fig 9 shows the same information as Fig 8, but depicted by site. Fig 9 emphasizes the additive (cumulative) effects of *Tones* and *Airguns*; within each site, whenever airguns were present, the predicted calling rates were consistently higher than when airguns were absent. Of course, within any one plot, the two lines are not parallel because the effects are additive on a log scale. While there is an apparent difference among sites (for example, the effect of *Airguns* appears to be greater at site 0 than at site 5), this is not indicative of an interaction between *Airguns* and *Tones*. Rather, it is a consequence of the interaction between *Airguns* and *Site* (as illustrated in Fig 6; see also interactions for top model in the S3 Table).

Calling rates were also calculated for two contrasting subsets of the observed data: CTIs that included both airguns and tones *versus* neither (all sites combined and excluding CTIs with only airguns or only tones). In this case, mean calling rates were 0.040 calls / CTI when neither industrial sound source was present, and 0.142 calls / CTI when both were detected—a 3.5-fold increase.

## Discussion

The primary result of this analysis is that whenever tones from industrial activity were present in the sound environment of bowhead whales during their fall migration, the whales’ calling rates were affected. With increasing dosages of “tone sound”, calling rates increased, peaked, and then decreased, all within the restricted broadband background levels (*BB* < 113 dB re 1 μPa) that were enforced in this analysis to avoid masking effects. This result parallels that from a previous study examining the effects of airgun pulses on bowhead calling rates [12], which showed that when subjected to sound from airgun pulses, bowhead calling rates similarly increased, plateaued, and then decreased after a certain threshold of sound was exceeded.

Observed mean calling rates in the absence and presence of the two sound sources give a sense of the scale of the increase. Mean calling rates, at all sites combined, increased by factors of 1.75 (0.056 to 0.098 calls per CTI) and 2.05 (0.044 to 0.090) when tones or airguns, respectively, were present in the background. In comparison, Blackwell *et al*. [12] reported an increase in calling rates by a factor of 1.7 when seismic operations were nearby (2007 and 2008 in [12]) and a smaller increase (x1.2) when seismic operations were distant (2009 and 2010).

The largest increase in calling rates occurred when tones and airguns happened concurrently, compared to times when neither were detected in the sound records: mean calling rates (calls per CTI for all sites combined) were 0.142 versus 0.040, respectively, a 3.55 factor difference. In addition the increase was site-dependent, due to the top model’s *Site* x *Airguns* interaction (Table 2), and roughly related to each site’s distance from *Sivulliq*. In Fig 9, the maximum predicted calling rates (“top of the mountain” of the model) at site 0 were 0.126 and 0.413 calls / CTI in the absence (dashed line) and presence (solid line) of airgun pulses, respectively. This corresponds to a 3.3 factor increase, the largest of any of the sites. The second largest increase was at site 4 (x2.3), followed by site 2 (x1.8), site 3 (x1.5), and the two most distant sites, sites 1 and 5 (x1.3). This relationship between the magnitude of the change in calling rate and the distance to *Sivulliq* is interesting, albeit somewhat surprising since not all the industrial activities were located at *Sivulliq*: there were several other sources of anthropogenic sound, *e*.*g*., the two seismic exploration operations (east and north of the DASARs) and the drilling-related vessel traffic that was almost entirely west of *Sivulliq* [3]. Nevertheless, authors have for some time suspected that context, such as the distance to a sound source, plays a role in how whales react to a particular received sound level [20]. We suspected such an effect when investigating the effects of airgun pulses on calling rates [12] and the findings presented here may be yet another example of the phenomenon. The nature of sound propagation in these shallow-water environments often embeds clues to the range of transient sounds, in the form of frequency-dependent dispersion that “stretches” the duration of a sound with increasing range. It would thus not be surprising if bowhead whales displayed an ability to infer the range of various sounds, and respond accordingly.

### Accounting for masking effects

The confounding of masking effects with behavioral changes is a common problem whenever calls are not localized and thus variations in call detectability cannot be taken into account [10]. We attempted to control for masking effects by removing periods with the highest broadband levels from the analysis. Ten-minute periods with mean broadband levels exceeding 113 dB re 1 μPa, representing 14% of all available samples, were excluded from the modeling. If we assume a source level of 161 dB re 1 μPa for bowhead calls [21] and a 15log(*R*) acoustic transmission loss (where *R* is range in meters between source and receiver), which is an appropriate acoustic propagation model for the study area [22, 21], the received level at 2 km is ~111 dB, a little below our received level cut-off. Nevertheless, we feel confident in our results for the following reasons: (1) the quantile regression in Fig 2 showed 2-km call localization rates to be independent from background levels whenever a 113 dB cut-off was used; (2) the results described here were unchanged when the analysis was run with a broadband cut-off at 110 dB; and (3) the main result, *i*.*e*., an *increase* in calling with an increase in noise levels, is the opposite trend from the decrease in calling one would expect if masking were a factor.

### Reasons for changes in calling rate

Whenever animal communication is hampered by noise, whether it be natural (*e*.*g*., increasing wind speeds in penguin colonies, [23]) or anthropogenic (*e*.*g*., plane overflights near amphibian colonies, [24]), there are three general strategies available to the communicator that wishes to maintain communication: increase the amplitude of the signal, change the frequency of the signal, or increase the repetition rate of the signal.

Increasing the amplitude of the signal—the so-called Lombard effect—has been observed in many vertebrates, see Brumm and Zollinger [25] for a review. Examples within marine mammals include right whales [26], killer whales [27], and beluga whales [28]. Changing the frequency of the signal—which often happens in concert with the change in amplitude described above [29, 30]—has been demonstrated in beluga whales [31] and right whales [32], as well as in bird species [33, 34]. Similarly, fin whales were found to modify the acoustic features of their calls when in the presence of sound sources such as vessels or airguns [35]. In the bowhead dataset presented here, little change in the frequency content of calls was found in response to the presence or absence of airgun activity [36].

The third strategy, to increase the repetition rate (redundancy) of the signal, is the strategy supported by the results presented here. Again, several examples exist from the bird literature [23, 37]. Amongst marine mammals, blue whales have been shown to increase their calling rate in response to seismic surveying [38], and humpback whales increase the rate and repetitiveness of feeding call types in the presence of vessel noise [39].

Both signal detection theory [40] and information theory [41, 39] demonstrate how increasing call repetition rates can be a viable strategy for combating decreased detectability of signals arising from moderate increases in background noise levels. This is likely the best explanation for increased calling rates in bowhead whales in response to increased doses of both airgun pulses [12] and tones (this study). Nevertheless, specific predictions from signal detection theory depend on assumptions about the detection thresholds (miss and false alarm rates) of listening whales, which are beyond this analysis. Also, the relevance of information theory to whale calling rates crucially depends on whether the rate of information production (“entropy rate,” with units of bits per second) generated by sequences of whale calls matches or exceeds the so-called “channel capacity” of an ocean acoustic waveguide, which is the maximum rate of information (in bits per second) that can be transmitted through a channel without incurring information loss. This assumption may be problematic, given that previous quantitative calculations of baleen whale entropy rates have yielded low values, on the order of a bit or less per second [39, 42]. These rates are well below the channel capacity of most ocean environments (as predicted by the Shannon–Hartley theorem, using the bandwidth and typical signal-to-noise ratio of whale calls).

Both signal detection theory and information theory predict that communication ceases whenever noise levels become so large that signals are almost completely masked—at that point no amount of repetition can succeed in increasing the probability of detection of a repeated sequence. Nevertheless, bowhead whales start decreasing their calling rates in response to higher doses of sound from airgun pulses [12] and tones (this study) at levels well below those of complete masking. It is possible that as noise increases, the benefits of calling decrease because information transfer becomes less effective (more error-prone) and is at some point no longer worth the effort, despite the fact that calls are not totally masked. To assess whether rising noise levels could lead to the loss of some part of a bowhead whale’s codified message, more knowledge is needed on the information content of calls as well as how this information content degrades in the presence of increasing noise levels of various types (*e*.*g*., continuous or intermittent, frequency range, etc.).

The automated call detection algorithm used in this analysis [16] did not perform call classification. If whales changed call type as a function of the type of anthropogenic sound they were subjected to, or as a function of overall calling rates [43], these changes would have been unnoticed in the analysis presented here. This is relevant because different call types have different dominant frequencies and durations, which could affect a whale’s communication range in a high-noise environment. Also note that the lack of call classification in this study also means that when the calling rate increases within a CTI, we do not know whether it’s the same type of call that is repeated.

In conclusion, little can be said at this point on the effects of changes in calling behavior on the long-term fitness of bowhead whales. The Bering-Chukchi-Beaufort population of bowhead whales has been increasing steadily over the last 30 years [44], despite episodic presence of industrial activities in their summering grounds and along their autumn migration route. Thus, the temporary effects on calling described here seem inconsequential in terms of population-level effects, but this assessment may need to be revisited if the level of industrial activities in the Beaufort Sea changes in the future.

## Supporting information

S1 File Calculation of the *Tones* index (with Figs A and B). (PDF)

S2 File Block bootstrapping. (PDF)

S1 Table Information relating to DASAR deployment locations. (PDF)

S2 Table Quantile regression estimates for lines shown in Fig 2 (regression models for the 97.5^th^ quantile). (PDF)

S3 Table Negative binomial regression models. (PDF)
