# Supplementary material for: Effects of tones associated with drilling activities on bowhead whale calling rates
Source: PLoS One. 2017 Nov 21;12(11):e0188459. doi: 10.1371/journal.pone.0188459 (PMC5697844; doi:10.1371/journal.pone.0188459)
Supplement: S1 File — (PDF) [file pone.0188459.s001.pdf]

S1 File. Section A (Calculation of the *Tones* index) and Figures A and B.

The *Tones* index quantifies the relative presence of tone-like structures in the sound spectrum, such as the 120 Hz hum from rectified power supplies, or other tones generated by rotating machinery. The basic concept behind the index is that it represents the relative amount of acoustic power present as tones in the background noise spectrum, with "tones" being defined here as stochastic narrowband noise whose statistics do not change over minute-long time scales. The *Tones* index was developed by trial and error, with several iterations of quality checking and adjustment. When assessing the effects of the Northstar oil production island on the southern edge of the bowhead whale migration past Prudhoe Bay, Alaska, McDonald *et al.* (2012) used a tone index (*ISI_tone*) that is similar, but not identical, to the one used in this study.

The computation of the index begins with two-min averaged spectra (10–450 Hz; 1-s samples, 50% data overlap) computed from the DASAR records, which are examined for the presence of tones. A tone was defined as present whenever the average power spectral density value (*single_tone_density)* for a given frequency was at least 4 dB (the *tone_threshold*) above the average level of the two frequency bins below and the two frequency bins above the frequency being examined. The average (in the linear domain) of those four neighboring frequency bins is defined here as the *local_background_level* (expressed in terms of dB). The *single_tone_value* is obtained by subtracting *local_background_level* from the *single_tone_density* (in the linear domain), and represents an estimate of the power spectral density arising from the tone only. Finally, to get the "total tone density" (*tone_density_level*) over the two-min period in question, all individual *single_tone_values* found in the two-min sample are summed using linear units before converting back into dB units.

Concrete example:

- Say the frequency of interest is 30 Hz. The *local_background_level* will be calculated from the values of the bins centered at 28, 29, 31, and 32 Hz (bins centered at 1 Hz intervals).
- If *single_tone_density* (30 Hz) is 102 dB and *local_background_level* is 97 dB (re 1 μPa^2 / Hz for both), then 102 – 97 is 5 dB, therefore the power at 30 Hz qualifies as a tone detection (because 5 dB > *tone_threshold* of 4 dB).
- Converting pressure into standard MKS units:

$10^{(102/10)}$ = 15,848,931,925 μPa^2 / Hz, and $10^{(97/10)}$ = 5,011,872,336 μPa^2 / Hz

- Subtracting one from the other:

15,848,931,925 – 5,011,872,336 = 10,837,059,588 μPa^2 / Hz. This *single_tone_value* is our estimate of the true power spectral density of the tone alone.

- This procedure is then applied for each tone identified in the 2-min sample. Say three tones are present in our 2-min data sample:

single_tone_value (30 Hz) = 10,837,059,588 μPa^2 / Hz (from above)

single_tone_value (60 Hz) = 9,222,359,844 μPa^2 / Hz

single_tone_value (130 Hz) = 11,684,119,945 μPa^2 / Hz

We sum all three above values: *tone_density* = 31,743,539,377 μPa^2 / Hz

Convert back to dB: *tone_density_level* = 10 $\log_{10}$(31,743,539,377) = 105.0 dB re 1 μPa^2 / Hz

We are interested in the amount by which tones protrude above background noise, whatever that background level may be, because it seems more likely that whales react to the signal-to-noise ratio of a tone, rather than the absolute power of the tone. In other words, a whale

may be more likely to react to a tone of amplitude X on a quiet day with low background levels, than to a tone of amplitude 2X that is barely above background because of high sea states. We therefore estimate something similar to a signal-to-noise ratio of the tone powers to mean background noise power, by converting the background sound pressure level *BB* (a band level in dB re 1 µPa, see body of paper) into an *adjusted_broadband_noise_level* (in dB re 1 μPa^2 / Hz). This is done by assuming the noise is fairly flat (white) and subtracting 10 $\log_{10}$(bandwidth). The bandwidth in all our recordings is 440 Hz (data range = 10–450 Hz), so 10 $\log_{10}(440)$ = 26.43 dB re 1 Hz. The *adjusted_broadband_noise_level* is then subtracted from the *tone_density_level* to yield $Tones_{Two\text{-}min}$, the total acoustic power from tones in the two-min sample. Finally, to get the value of *Tones* for an entire 10-min cell-time interval, the five $Tones_{Two\text{-}min}$ values are summed in the linear domain and then converted back to dB.

Returning to our example:

- If *BB* (broadband background level, see body of paper) is 122.5 dB re 1 μPa, then:

  adjusted_broadband_noise_level = 122.5 – 26.43 = 96.1 dB re 1 μPa^2 / Hz

  $Tones_{Two\text{-}min}$ = *tone_density_level* – *adjusted_broadband_noise_level* = 105.0 – 96.1 = 8.9 dB

- Say the five $Tones_{Two\text{-}min}$ values included in a 10-min cell-time interval (CTI) are 8.9 dB, 12.2 dB, 11.2 dB, 7.4 dB, and 11.2 dB, then the *Tones* value for that 10-min CTI is 17.5 dB (values converted into standard MKS units, as described above, added, and converted back to dB).

Tones can take on both positive and negative values. Positive values are what we would expect, but negative values can occur due to the fact that in computing the *adjusted_broadband_noise_level*, we simply use the average spectral density level corresponding to a broadband level (by subtracting 26.43 dB). If the detected tones are at frequencies different from where there is a peak in sound energy, the average spectral density level that is subtracted from the *tone_density_level* is too large, and we may end up with negative *Tones* values. Regardless, tones **were** still detected in those samples, so they still serve their role as an index. Therefore, once the *Tones* value was calculated for every cell-time interval at every DASAR, an arbitrary offset of 32 dB was added to every *Tones* value, to translate all *Tones* values into the positive domain. Fig A shows a bar graph of the *Tones* values obtained over the entire season at each site.

The most severe problem encountered by early versions of the *Tones* index was contamination by airgun signals. This happened because a repetitive transient signal (such as an airgun pulse) can generate peaks in an averaged spectrum that could artificially trigger the tones detector—an obviously unacceptable situation for the analysis presented here. Considering that the exact time of each airgun pulse had been identified by an airgun pulse detector (Thode *et al.* 2010), a first step in dealing with the contamination problem was simply to excise the airgun pulses (pulse duration ± 0.5 s on each side) out of the datasets and run the tone detector on the remaining data. (Thus the initial 2-min data sample was actually reduced to a shorter time period, once airgun signals were excised.) The tones we wished to detect were expected to occur over durations lasting from minutes to hours, and would thus be unaffected by the excisions.

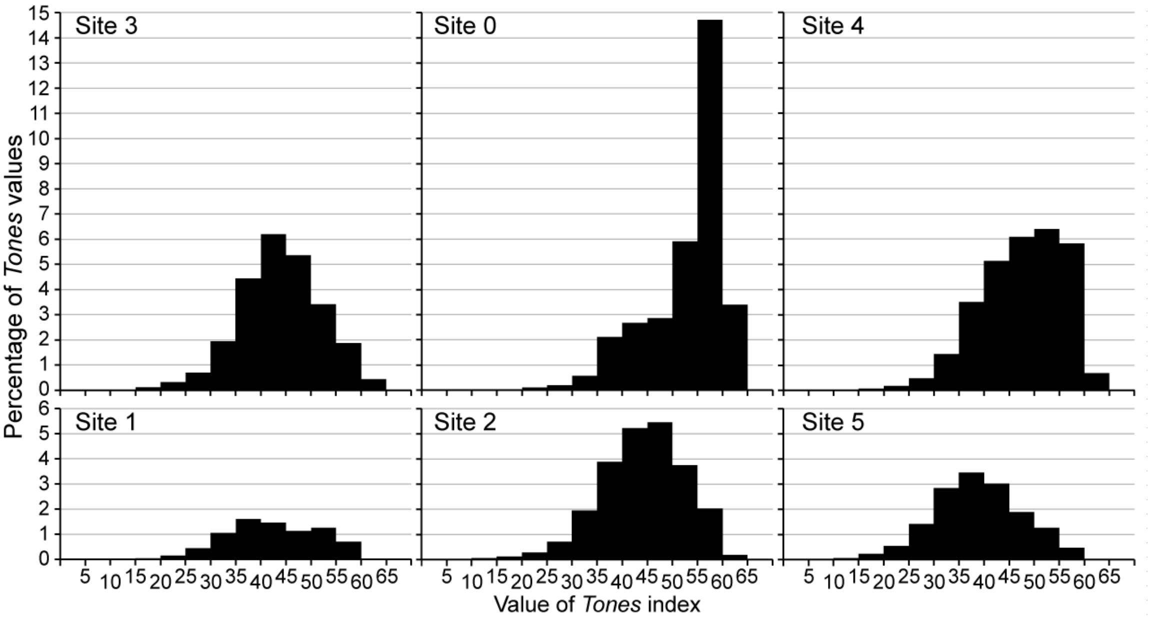

Fig A. Histograms of the values of the *Tones* index at each site over the entire season. Sites on the top row are those close to the drilling operations (sites 3, 0, and 4) and those on the bottom row were farther away (sites 1, 2, and 5). For clarity, samples with *Tones* = 0 are not shown, but contributed the following percentages: 92% for Site 1, 76% for Site 2, 75% for Site 3, 67% for site 0, 70% for site 4, and 85% for site 5.

Quality checking of the detections—*i.e.*, making sure airgun pulses were not contaminating the *Tones* detections—were done using the particle velocity data collected by the DASARs. We knew the locations of the sources of airgun pulses because the airgun pulse detector (Thode *et al.* 2010) outputs the bearing to each detected pulse. We also had position information for the vessels involved in Shell's drilling operation most of the time. Using this knowledge, we could select times at particular DASARs when tone-producing activities and airgun pulses were occurring concurrently but in different directions, and check whether the bearings given were independent (*i.e.*, pointing in different directions). Fig B shows some of these bearing plots for one particular DASAR location (4L) and how they were used to refine the *Tones* detector. The results show that an explicit removal of airgun signals, combined with a 4 dB *tone_threshold* and 113 broadband noise cutoff, was sufficient to remove all influence by airgun surveys.

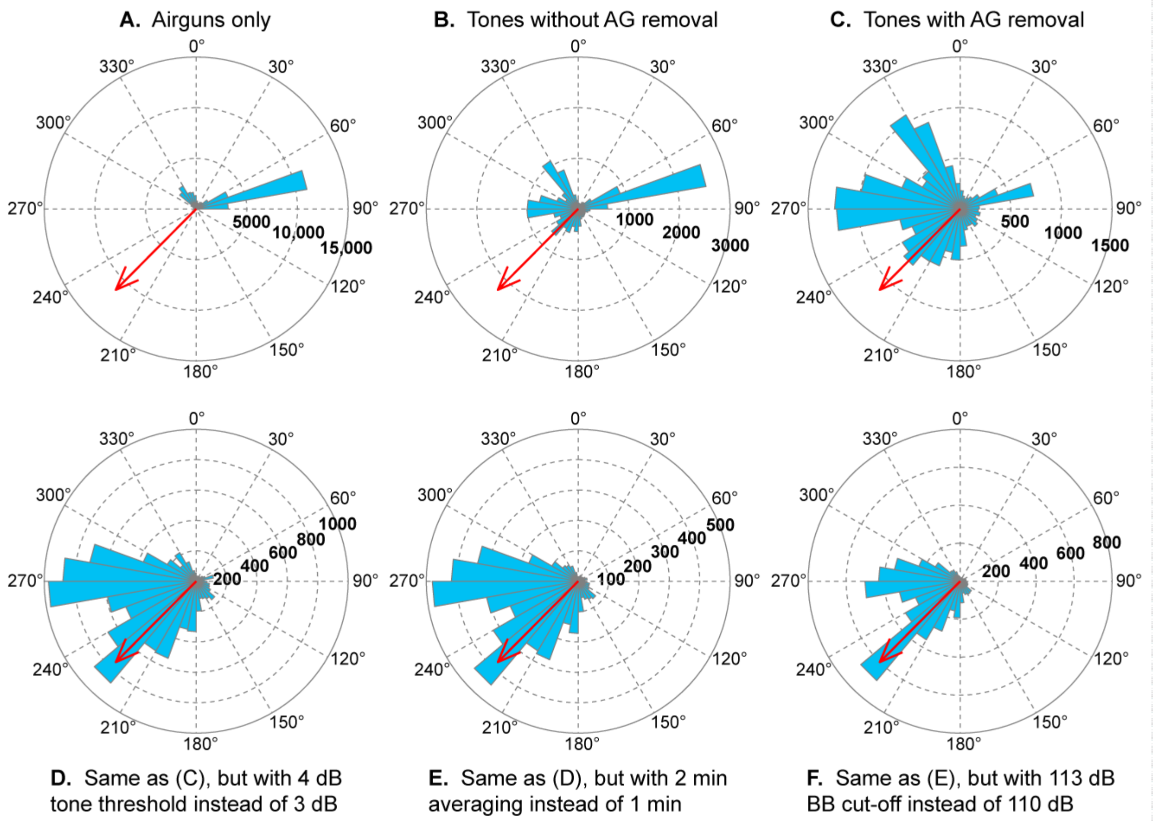

Fig B. Rose plots of bearings showing how the choice of various parameters affected the detections of tones at DASAR 4L over the entire 2012 deployment season. The red arrow in each plot points towards the *Sivulliq* drilling location. AG = airgun, BB = broadband. **(A)** Bearings to airgun pulses as detected by the airgun pulse detection code. Two main bearing directions are shown: ENE and NNW. **(B)** Bearings to detected tones (3 dB threshold, 1-min averaging, 110 dB cut-off, see text for more information) without prior airgun (AG) removal first. The tone bearings are dominated by detections to the two seismic operations (ENE and NNW), because of contamination by airgun pulses. **(C)** Bearings to detected tones (3 dB threshold, 1-min averaging, 110 dB cut-off) **with** prior AG removal first. The relative importance of the bearings pointing towards the seismic operations (ENE and NNW) has decreased. **(D)** Bearings to detected tones (**4 dB threshold**, 1-min averaging, 110 dB cut-off) with prior AG removal first. Increasing the tone detection threshold from 3 dB to 4 dB has again decreased the number of false detections due to airguns. **(E)** Bearings to detected tones (4 dB threshold, **2-min averaging**, 110 dB cut-off) with prior AG removal first. Increasing the time over which the data are averaged for tone detection from 1 min to 2 min has again decreased the number of false detections due to airguns. **(F)** Bearings to detected tones (4 dB threshold, 1-min averaging, **113 dB cut-off**) with prior AG removal first. Including time intervals with an average broadband level of up to 113 dB, instead of 110 dB, increased the detection of tones from *Sivulliq*, as shown by the increased relative number of detections from that direction (220°–230°).

McDonald TL, Richardson WJ, Greene CR Jr, Blackwell SB, Nations CS, Nielson RM, *et al.* Detecting changes in distribution of calling bowhead whales exposed to fluctuating anthropogenic sounds. J Cetacean Res Manag. 2012; 12:91–106.

Thode A, Kim KH, Greene CR Jr, Roth E. Long range transmission loss of broadband seismic pulses in the Arctic under ice-free conditions. J Acoust Soc Am. 2010; 128:EL181–EL187.
