# Supplementary material for: Effects of tones associated with drilling activities on bowhead whale calling rates
Source: PLoS One. 2017 Nov 21;12(11):e0188459. doi: 10.1371/journal.pone.0188459 (PMC5697844; doi:10.1371/journal.pone.0188459)
Supplement: S2 File — (PDF) [file pone.0188459.s002.pdf]

S2 File. Block bootstrapping.

The bootstrap procedure was carried out as follows. (1) For each bootstrap iteration a starting time was randomly generated within the 160-min period preceding the first cell-time interval. (2) Sequential non-overlapping 160-min blocks of time were defined such that all observations were assigned to a time block. (3) For each site, a new time series was constructed by sampling time blocks with replacement. (4) The negative binomial regression model was re-fitted to the iteration dataset, and the resulting parameter estimates were stored. (5) One thousand block bootstrap iterations were completed to compute the distribution means, and the 95% confidence intervals using the percentile method.

Because previous analyses (McDonald *et al.* 2012, Blackwell *et al.* 2015) have indicated that autocorrelation in calling rates may exist, we examined the data for both temporal and spatial autocorrelation.

Time. To determine the length of time over which autocorrelation may occur, we examined partial autocorrelation functions (PACFs). The PACF was considered informative for our purposes because it discounts the carryover effect of lower-order autocorrelations within the higher-order autocorrelations. (Typically, the PACF is used to judge the appropriate order of autoregressive models in time series analysis.) Because the appropriate lag appeared to vary considerably by DASAR, we examined the PACF of the combined data by averaging the estimated PACFs across DASARs. This exercise indicated that a lag of 16 intervals (160 min) was an appropriate time block length.

Space. To determine the potential distance over which spatial autocorrelation may be occurring, we estimated Moran's I (1950) for residuals from groups of DASARs within varying distance bands. Because the magnitude of Moran's I alone does not provide insight into whether spatial autocorrelation exists, we examined the proportion of Moran's I p-values that were significant between DASARs within 8 and 16 km of one another. This exercise indicated that spatial autocorrelation within sites was not a problem.

Blackwell SB, Nations CS, McDonald TL, Thode AM, Mathias D, Kim KH, *et al.* Effects of airgun sounds on bowhead whale calling rates: evidence for two behavioral thresholds. PLoS ONE 2015; 10(6):e0125720.

McDonald TL, Richardson WJ, Greene CR Jr, Blackwell SB, Nations CS, Nielson RM, *et al.* Detecting changes in distribution of calling bowhead whales exposed to fluctuating anthropogenic sounds. J Cetacean Res Manag. 2012; 12:91–106.

Moran, PAP. Notes on continuous stochastic phenomena. Biometrika. 1950; 37: 17–23.
