# Supplementary material for: Effects of tones associated with drilling activities on bowhead whale calling rates
Source: PLoS One. 2017 Nov 21;12(11):e0188459. doi: 10.1371/journal.pone.0188459 (PMC5697844; doi:10.1371/journal.pone.0188459)
Supplement: S1 Table — (PDF) [file pone.0188459.s003.pdf]

**S1 Table. Information relating to DASAR deployments.** Deployment coordinates (decimal degrees, WGS 84), deployment and retrieval dates in 2012, and water depth for all DASAR locations.

| Site | Position | Lat. (°N) | Long. (°W) | Depth (m) | Deployment | Retrieval |
|---|---|---|---|---|---|---|
| 1 | D | 70.8864 | 150.4903 | 23.7 | 21 Aug | 3 Oct |
| 1 | E | 70.9146 | 150.6444 | 20.6 | 21 Aug | 3 Oct |
| 1 | F | 70.9491 | 150.4908 | 22.6 | 21 Aug | 3 Oct |
| 2 | A | 70.6383 | 148.9512 | 18.6 | 18 Aug | 4 Oct |
| 2 | B | 70.6696 | 148.7869 | 21.8 | 18 Aug | 4 Oct |
| 2 | C | 70.7015 | 148.9511 | 24.4 | 18 Aug | 4 Oct |
| 2 | D | 70.7324 | 148.7869 | 26.9 | 18 Aug | 4 Oct |
| 2 | E | 70.7638 | 148.9513 | 25.5 | 18 Aug | 4 Oct |
| 2 | F | 70.7952 | 148.7865 | 32.6 | 18 Aug | 4 Oct |
| 2 | G | 70.8267 | 148.9516 | 32.0 | 18 Aug | 4 Oct |
| 3 | A | 70.3866 | 146.8030 | 28.1 | 19 Aug | 7 Oct |
| 3 | B | 70.4180 | 146.6411 | 32.6 | 19 Aug | 7 Oct |
| 3 | C | 70.4495 | 146.8030 | 32.4 | 19 Aug | 7 Oct |
| 3 | D | 70.4808 | 146.6402 | 37.7 | 19 Aug | 7 Oct |
| 3 | E | 70.5123 | 146.8035 | 37.9 | 19 Aug | 6 Oct |
| 3 | F | 70.5435 | 146.6399 | 38.2 | 19 Aug | 6 Oct |
| 3 | G | 70.5750 | 146.8035 | 38.9 | 19 Aug | 6 Oct |
| 4 | AA | 70.2484 | 145.4002 | 29.4 | 17 Aug | 6 Oct |
| 4 | B | 70.2824 | 145.5599 | 30.6 | 17 Aug | 6 Oct |
| 4 | CC | 70.3115 | 145.3990 | 34.7 | 17 Aug | 6 Oct |
| 4 | D | 70.3452 | 145.5596 | 33.0 | 17 Aug | 6 Oct |
| 4 | EE | 70.3742 | 145.3975 | 37.6 | 17 Aug | 6 Oct |
| 4 | F | 70.4082 | 145.5583 | 39.4 | 17 Aug | 6 Oct |
| 4 | GG | 70.4374 | 145.3970 | 40.0 | 17 Aug | 6 Oct |
| 4 | H | 70.4082 | 145.8824 | 36.3 | 16 Aug | 7 Oct |
| 4 | I | 70.3455 | 145.8822 | 32.2 | 16 Aug | 7 Oct |
| 4 | J | 70.4713 | 145.5523 | 38.2 | 17 Aug | 6 Oct |
| 4 | K | 70.5005 | 145.3878 | 45.3 | 17 Aug | 6 Oct |
| 4 | L | 70.5334 | 145.5431 | 37.4 | 17 Aug | 6 Oct |
| 4 | M | 70.5633 | 145.3809 | 54.4 | 17 Aug | 6 Oct |
| 0 | X | 70.4166 | 146.1238 | 35.7 | 16 Aug | 7 Oct |
| 0 | Y | 70.4120 | 146.1754 | 35.3 | 16 Aug | 7 Oct |
| 0 | Z | 70.4296 | 146.1611 | 35.9 | 16 Aug | 7 Oct |

**(S1 Table continued)**

| Site | Position | Lat. (°N) | Long. (°W) | Depth (m) | Deployment | Retrieval |
|---|---|---|---|---|---|---|
| 5 | A | 70.2466 | 143.3152 | 38.4 | 20 Aug | 5 Oct |
| 5 | B | 70.2781 | 143.1540 | 43.4 | 20 Aug | 5 Oct |
| 5 | C | 70.3093 | 143.3147 | 44.9 | 20 Aug | 5 Oct |
| 5 | D | 70.3411 | 143.1538 | 52.0 | 20 Aug | 5 Oct |
| 5 | E | 70.3721 | 143.3150 | 52.6 | 20 Aug | 5 Oct |
| 5 | F | 70.4038 | 143.1533 | 51.6 | 20 Aug | 5 Oct |
| 5 | G | 70.4355 | 143.3151 | 51.9 | 20 Aug | 5 Oct |
