# Supplementary material for: Effects of tones associated with drilling activities on bowhead whale calling rates
Source: PLoS One. 2017 Nov 21;12(11):e0188459. doi: 10.1371/journal.pone.0188459 (PMC5697844; doi:10.1371/journal.pone.0188459)
Supplement: S2 Table — (PDF) [file pone.0188459.s004.pdf]

S2 Table. Quantile regression estimates for lines shown in Fig 2 (regression models for the 97.5th quantile). Point estimates and lower and upper 95% confidence limits (L95 and U95, respectively), for the intercepts and slopes for four cut-off values (107 dB, 110 dB, 113 dB, and 116 dB), two datasets (**A.** All call localizations, and **B.** Only call localizations within 2 km of a DASAR), and two lower cut-offs (100 dB and 95 dB). See text for more information.

**A. All Call Localizations**				
	**Intercept**		**Slope**	
	**Estimate**	**L97.5, U97.5**	**Estimate**	**L95, U95**
A.1. Lowest cut-off set at 100 dB				
107 dB	191.09	176.45, 210.83	-1.70	-1.89, -1.56
110 dB	175.66	167.22, 187.99	-1.55	-1.67, -1.47
113 dB	164.51	156.38, 170.91	-1.45	-1.50, -1.37
116 dB	137.55	133.99, 140.57	-1.19	-1.21, -1.16
A.2. Lowest cut-off set at 95 dB				
107 dB	196.87	188.14, 206.11	-1.76	-1.85, -1.67
110 dB	188.15	181.53, 194.07	-1.67	-1.73, -1.61
113 dB	176.90	172.78, 180.89	-1.56	-1.60, -1.52
116 dB	150.38	148.05, 152.79	-1.30	-1.32, -1.28

**B. Only Call Localizations Within 2 km of a DASAR**				
	**Intercept**		**Slope**	
	**Estimate**	**L97.5, U97.5**	**Estimate**	**L97.5, U97.5**
B.1. Lowest cut-off set at 100 dB				
107 dB	1	1, 1	0	0, 0 *
110 dB	1	1, 1	0	0, 0 *
113 dB	1	1, 10.96	0	-0.097, 0
116 dB	8.65	8.50, 8.79	-0.075	-0.076, -0.073
B.2. Lowest cut-off set at 95 dB				
107 dB	1	1, 1	0	0, 0 *
110 dB	1	1, 1	0	0, 0 *
113 dB	1	1, 1	0	0, 0 *
116 dB	1	1, 1	0	0, 0 *

* Intercept = 1 and Slope = 0 in all 1000 bootstrap iterations
