# Supplementary material for: Effects of tones associated with drilling activities on bowhead whale calling rates
Source: PLoS One. 2017 Nov 21;12(11):e0188459. doi: 10.1371/journal.pone.0188459 (PMC5697844; doi:10.1371/journal.pone.0188459)
Supplement: S3 Table — (PDF) [file pone.0188459.s005.pdf]

**S3 Table. Negative binomial regression models.** Candidate models, ranked by BIC. np = number of parameters in model. Within the *Effects* column interactions are shown within parentheses, and an exponent within parentheses implies inclusion of lower-order terms, *e.g.*, $Tones^{(2)}$ is equivalent to $Tones + Tones^2$, and ($Site \times Tones^{(2)}$) is equivalent to ($Site \times Tones$) + ($Site \times Tones^2$).

| Rank | BIC | np | Effects |
|---|---|---|---|
| 1 | 93515 | 15 | *Airguns* + $Tones^{(3)}$ + *Site* + (*Site* x *Airguns*) |
| 2 | 93531 | 10 | *Airguns* + $Tones^{(3)}$ + *Site* |
| 3 | 93633 | 30 | *Airguns* + $Tones^{(3)}$ + *Site* + (*Site* x *Airguns*) + (*Site* x $Tones^{(3)}$) |
| 4 | 93645 | 25 | *Airguns* + $Tones^{(3)}$ + *Site* + (*Site* x $Tones^{(3)}$) |
| 5 | 93720 | 14 | *Airguns* + $Tones^{(2)}$ + *Site* + (*Site* x *Airguns*) |
| 6 | 93744 | 9 | *Airguns* + $Tones^{(2)}$ + *Site* |
| 7 | 93762 | 24 | *Airguns* + $Tones^{(2)}$ + *Site* + (*Site* x *Airguns*) + (*Site* x $Tones^{(2)}$) |
| 8 | 93775 | 19 | *Airguns* + $Tones^{(2)}$ + *Site* + (*Site* x $Tones^{(2)}$) |
| 9 | 93972 | 9 | $Tones^{(3)}$ + *Site* |
| 10 | 94031 | 5 | *Airguns* + $Tones^{(3)}$ |
| 11 | 94088 | 24 | $Tones^{(3)}$ + *Site* + (*Site* x $Tones^{(3)}$) |
| 12 | 94185 | 8 | $Tones^{(2)}$ + *Site* |
| 13 | 94208 | 18 | $Tones^{(2)}$ + *Site* + (*Site* x $Tones^{(2)}$) |
| 14 | 94235 | 4 | *Airguns* + $Tones^{(2)}$ |
| 15 | 94389 | 18 | *Airguns* + *Tones* + *Site* + (*Site* x *Airguns*) + (*Site* x *Tones*) |
| 16 | 94429 | 13 | *Airguns* + *Tones* + *Site* + (*Site* x *Tones*) |
| 17 | 94506 | 13 | *Airguns* + *Tones* + *Site* + (*Site* x *Airguns*) |
| 18 | 94562 | 8 | *Airguns* + *Tones* + *Site* |
| 19 | 94665 | 4 | $Tones^{(3)}$ |
| 20 | 94762 | 12 | *Airguns* + *Site* + (*Site* x *Airguns*) |
| 21 | 94793 | 7 | *Airguns* + *Site* |
| 22 | 94855 | 3 | $Tones^{(2)}$ |
| 23 | 94985 | 12 | *Tones* + *Site* + (*Site* x *Tones*) |
| 24 | 95141 | 7 | *Tones* + *Site* |
| 25 | 95176 | 3 | *Airguns* + *Tones* |
| 26 | 95341 | 2 | *Airguns* |
| 27 | 95383 | 6 | *Site* |
| 28 | 95979 | 2 | *Tones* |
